# Supplementary material for: Predicting the Future Geographic Distribution of the Traditional Chinese Medicinal Plant Epimedium acuminatum Franch. in China Using Ensemble Models Based on Biomod2
Source: Plants (Basel). 2025 Mar 30;14(7):1065. doi: 10.3390/plants14071065 (PMC11990661; doi:10.3390/plants14071065)
Supplement: Supplementary file 1 [file plants-14-01065-s001.zip › Table S1.pdf]

Table S1. The KAPPA, TSS, and AUC values generated by the two ensemble models (EMca and EMwmean).

Abbreviation	EMca	EMwmean
TSS	0.931	0.930
Kappa	0.866	0.757
AUC	0.992	0.990
