# Supplementary figures and images for: Predicting the Future Geographic Distribution of the Traditional Chinese Medicinal Plant Epimedium acuminatum Franch. in China Using Ensemble Models Based on Biomod2
Source: Plants (Basel). 2025 Mar 30;14(7):1065. doi: 10.3390/plants14071065 (PMC11990661; doi:10.3390/plants14071065)

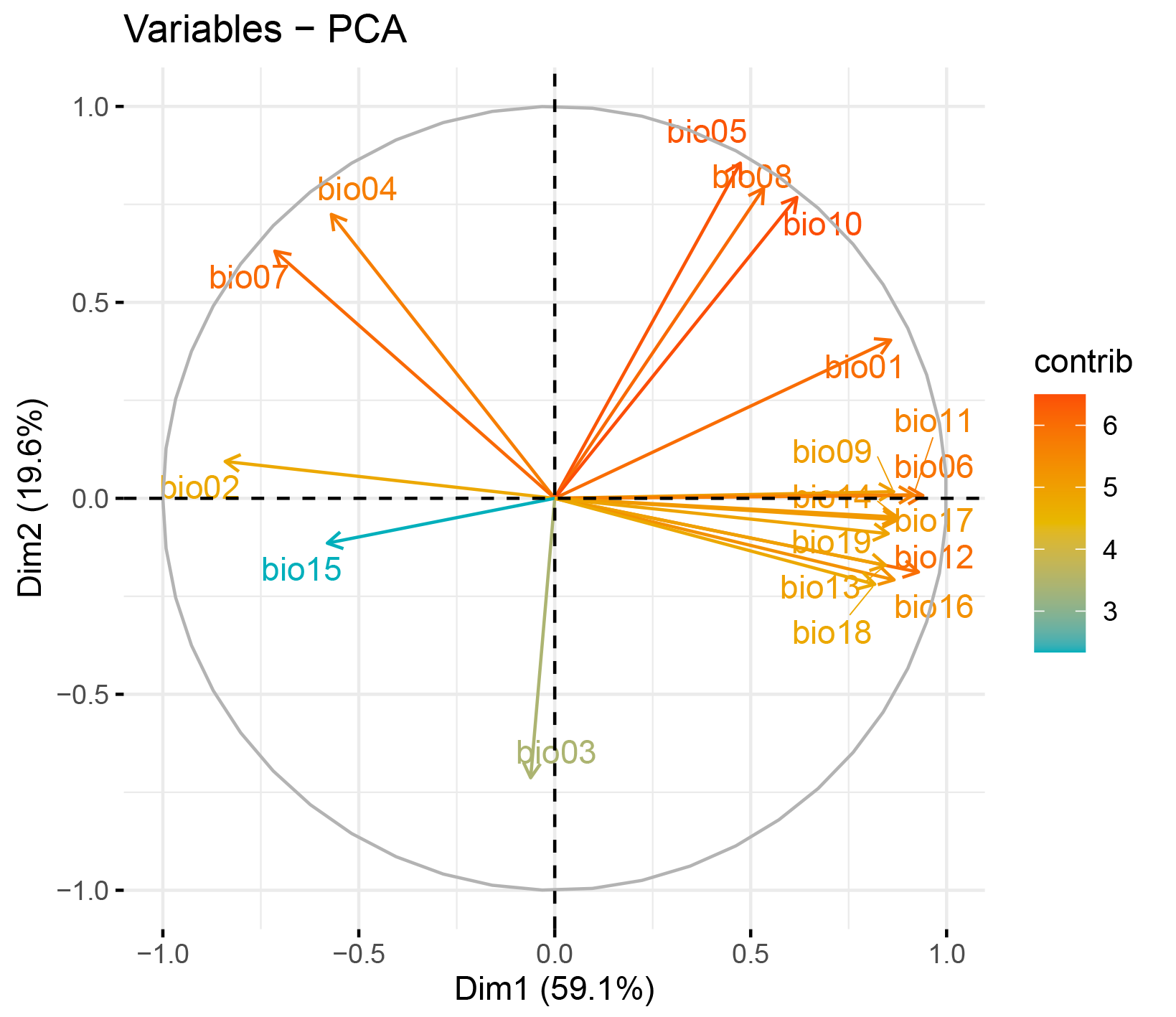

Supplement: Supplementary file 1 [file plants-14-01065-s001.zip › Figure S1.tif]
